# Transient appearance of circulating tumor DNA associated with *de novo* treatment

**DOI:** 10.1038/srep38639

**Published:** 2016-12-09

**Authors:** Kikuya Kato, Junji Uchida, Yoji Kukita, Toru Kumagai, Kazumi Nishino, Takako Inoue, Madoka Kimura, Fumio Imamura

**Affiliations:** 1 Department of Molecular and Medical Genetics, Research Institute, Osaka Medical Center for Cancer and Cardiovascular Diseases, Osaka, Japan; 2 Department of Thoracic Oncology, Osaka Medical Center for Cancer and Cardiovascular Diseases, Osaka, Japan

## Abstract

The limitation of circulating tumor DNA (ctDNA) is its inability to detect cancer cell subpopulations with few or no dying cells. Lung cancer patients subjected to the EGFR tyrosine kinase inhibitor (EGFR-TKI) treatment were prospectively collected, and ctDNA levels represented by the activating and T790M mutations were measured. The first data set (21 patients) consisting of samples collected in the period from before initiation of EGFR-TKI to at least 2 weeks after initiation: the ctDNA dynamics generally exhibited a rapid decrease and/or a transient increase. In 4 patients, we detected a transient increase of ctDNA bearing activating mutations not identified in biopsy samples. ctDNA with the same genotypical pattern was identified in 7 out of the 39 patients of the second data set intended to include samples until the onset of disease progression. In 6 of the 7 patients, this unique ctDNA appeared in the early period after treatment initiation, and did not reappear even after disease progression or chemotherapy. In another patient, similar ctDNA appeared upon radiation therapy. The identification of ctDNA with a unique genotype indicates the presence of cancer cell subpopulations that normally contain few or no dying cells, but generate dead cells because of the treatment.

Circulating tumor DNA (ctDNA) is the cell-free DNA that is released from dying or dead cancer cells into the blood stream^1^. As there are no mechanisms to release nuclear DNA from living cells, the death of cancer cells is a prerequisite for the generation of ctDNA. In addition, many studies have revealed a correlation between tumor burden and the ctDNA levels^2^, suggesting that a certain proportion of the cancer cell population is constantly dying. ctDNA normally represents cancer cell populations that actively proliferate and continuously renew themselves.

However, there are cancer cell populations with inconsistent or very low proportions of dying cells, in which the ctDNA levels do not reflect the tumor burden or are lower than the detection limit. These populations could be dormant, i.e., their cells are in a non-dividing state and their physiological functions become paused or quiescent^3^. As dormant cell populations are bound to release limited amounts of ctDNA, the presence of such populations cannot be detected through ctDNA analysis.

We have been studying the dynamics of ctDNA by monitoring ctDNA levels in lung cancer patients undergoing EGFR-TKI treatment^4^,^5^. We discovered ctDNA with a genotype that was absent from the corresponding biopsy samples. This ctDNA made its appearance in the early stages of the EGFR-TKI treatment, then disappeared and did not reappear during the observation time, including time after disease progression. We also discovered a unique-genotype ctDNA in cancer patients receiving radiation treatment, with its appearance coinciding with the therapy. The cancer cell subpopulations that are represented by such ctDNA usually do not contain dying cells, but rather generate dead cells as a consequence of therapy-induced cell destruction.

## Results

### Patients exhibited heterogenous ctDNA dynamics during the early period of EGFR-TKI treatment

**Figure 1:**
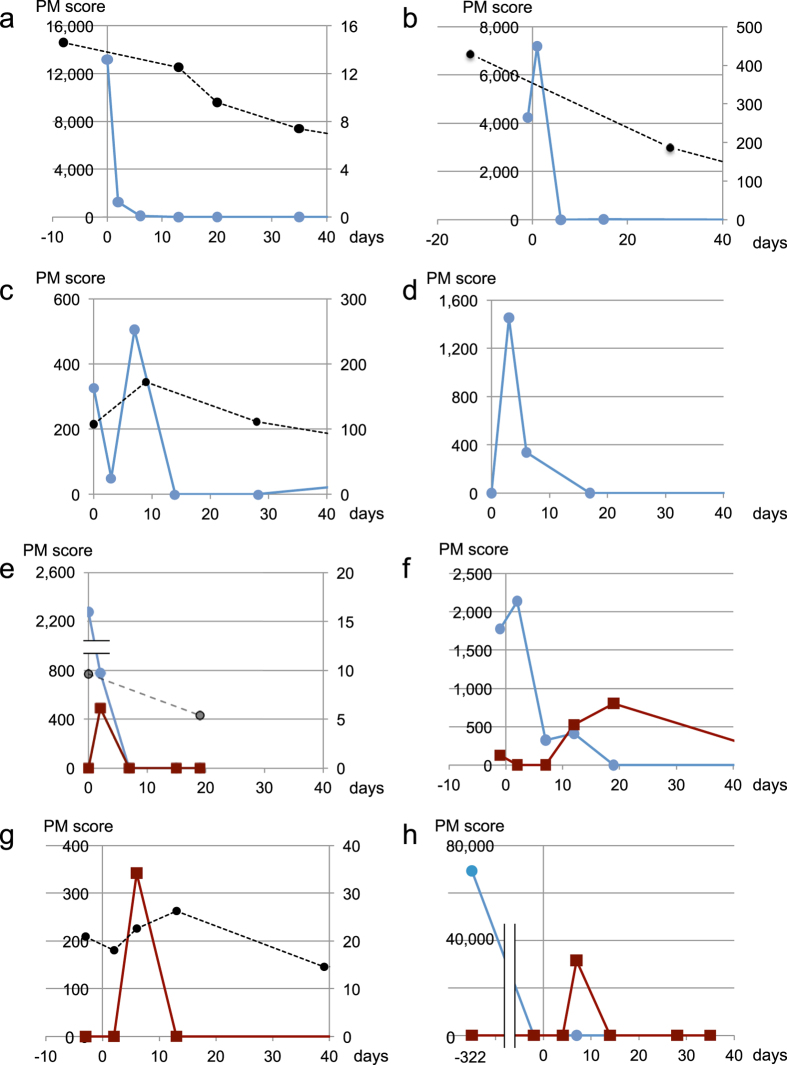
ctDNA dynamics in the early period of EGFR-TKI treatment. The vertical axis displays the PM score for *EGFR* mutations (left) or the concentration (μg/mL) of the carcinoembryonic antigen (CEA) (right). The horizontal axis displays the number of days after the initiation of the EGFR-TKI treatment. Blue lines indicate ctDNA with activating mutations identified in biopsy samples (exon 19 deletion or L858R). Wine red lines represent activating mutations absent from biopsy samples. Black broken lines represent CEA values. PM scores that did not exceed LOD, and CEA scores lying within the normal range are not displayed in the graphs. (**a**) Patient E1; (**b**) Patient E2; (**c**) Patient E3; (**d**) Patient E4; (**e**) Patient E5; (**f**) Patient E6; (**g**) Patient E7; (**h**) Patient E8. Clinical information on patients E1-E4, (age, sex, stage, EGFR-TKI, initial response, mutations in biopsy samples) are as follows: E1 (81, F, IV, erlotinib, PR, L858R); E2 (54, F, IV, gefitinib, PR, L858R); E3 (69, M, IV, gefitinib, PR, exon 19 deletion); E4 (56, F, IIA, gefitinib, NE, exon 19 deletion). Information on patients E5-E8 is presented in Table 1.

Analysis of the first data set, i.e., the one created for early response analysis, revealed that 14 out of the 21 patients had a ctDNA-positive sample prior to the EGFR-TKI treatment, whereas a total of 18 patients presented at least one positive sample during monitoring (before and/or after treatment). All data on ctDNA dynamics are graphically depicted in Fig. 1 and Supplementary Figure S1. Patient information is summarized in Supplementary Table S1. Patients exhibited a considerable variation in ctDNA dynamics. The levels of ctDNA in patient E1 (Fig. 1a) were high before EGFR-TKI treatment, but sharply decreased after the initiation of the treatment. Patients E2 and E3 (Fig. 1b and c, respectively) exhibited a transient increase in ctDNA levels observed at samples collected at day 1 or 7, respectively, and then sharply decreased. These transient increases probably represent release of nuclear DNA from cancer cells destroyed by the EGFR-TKI treatment. In patient E4 (Fig. 1d), no ctDNA was detected prior to treatment; however, an increase was observed in the sample collected on the third day of treatment. The ctDNA levels had largely stabilized after 2 weeks. Although there were atypical cases, the early ctDNA dynamics were generally characterized by a treatment-induced rapid decrease and/or transient increase.

### Transient appearance of ctDNA with activating mutations not identified in biopsy samples

In 4 patients, we detected ctDNA bearing activating mutations that were not identified in biopsy samples. This type of ctDNA appeared during the early period of the EGFR-TKI treatment and then disappeared, whereas it did not reappear during the observation period (Fig. 1e–h, Table 1). The presence of such ctDNA suggested the presence of cancer cell subpopulations with genotypes different from those detected in biopsy samples. We coined the term “T-type ctDNA” to define ctDNA transiently appearing upon initiation of new therapeutic protocols such as EGFR-TKI treatment. In cases with a T-type ctDNA whose genotype is identical to that in biopsy samples, the source cancer cell subpopulation cannot be distinguished from that of conventional ctDNA by means of ctDNA genotyping. Thus, we focused on identifying T-type ctDNA with a genotype that is absent from the corresponding biopsy samples. Patient E8 deserves special mention (Fig. 1h) because she had undergone both chemoradiation therapy and focal brain irradiation therapy, 10 months and 1 month before being subjected to erlotinib, respectively. High levels of ctDNA with the exon 19 deletion were observed before the chemoradiation therapy. This type of ctDNA had disappeared before the initiation of the EGFR-TKI treatment. After the initiation of erlotinib administration, the exon 19 deletion did not reappear; instead, ctDNA with L858R was detected. This suggests that erlotinib targeted lesions different from the primary lesion; the cells bearing the exon 19 deletion had been destroyed by the chemoradiation therapy.

### ctDNA with activating mutations not identified in biopsy samples was not detected during the later period of disease course

We scanned the second dataset, which contained samples from long-term observations, for ctDNA with activating mutations not identified in the biopsy samples. The data set consisted of 371 samples collected from 39 patients (sampling time intervals: median = 49 days, lower quartile = 28 days, upper quartile = 63 days). The data points were classified into three groups. The first corresponded to the early period of the EGFR-TKI treatment, including the first and the second assays (i.e. until approximately one month after the initiation of the EGFR-TKI treatment). The second group included the samples that were collected afterwards and until the onset of objective disease progression, whereas the third group contained the samples that were collected after the onset of disease progression. The numbers of data points included in the three groups were 68, 161, and 142, respectively.

**Figure 2:**
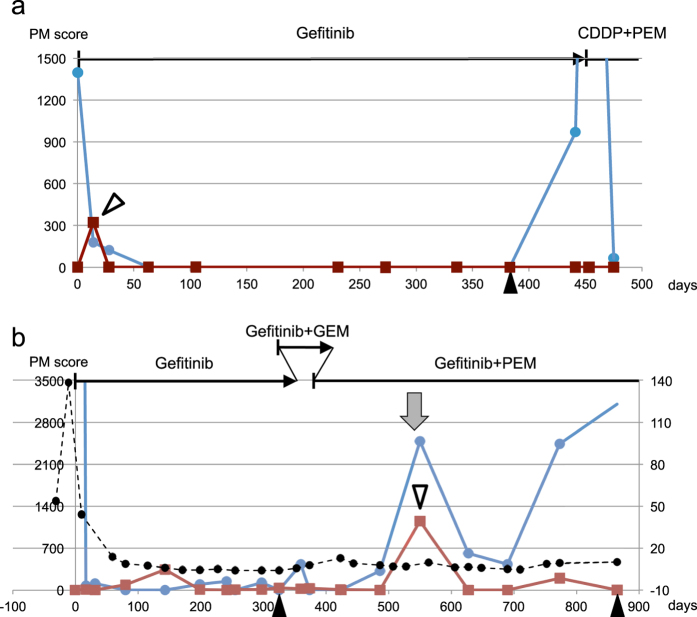
ctDNA dynamics of patients during long observation periods. (**a**) Patient 5; (**b**) Patient 10; Black arrowheads indicate the onset of objective disease progression. The white arrowhead indicates transient increase described in the main text. Red lines indicate ctDNA with T790M. The gray arrow indicates radiotherapy. Horizontal lines at the top of each panel indicate treatment, vertical bars indicate initiation of therapy, and arrowheads indicate termination of therapy. Other details are the same as those of Fig. 1. GEM, gemcitabine.

Thus, we found 7 ctDNA-positive samples from 7 patients (Table 1). 6 data points appeared in the early period, whereas one positive sample appeared in the period until disease progression (patient 3). Segregation of the ctDNA in the early period was statistically significant (Fischer’s exact test, p = 0.0002). In these 6 patients, the detected ctDNA was classified as T-type, as it appeared once, then disappeared and did not reappear during the observation time. It is important to note that the T-type ctDNA did not appear during disease progression (5 out of the 6 patients), or after chemotherapy (patients 5 and 15). Figure 2a depicts the data points from a representative patient (patient 5).

The false-positive rate of our assay system was estimated at 0–2% based on the diagnostic specificities^6^. This represents the maximum estimate, and the actual rate is likely lower because the calculation of the specificities was based on the assumption that there were no false negatives from the biopsy samples. In patients E5, E8, 2, and 15 the corresponding peaks were confirmed using deep sequencing with/without the molecular barcode.

### Radiation therapy-induced destruction of cancer cells leads to the appearance of ctDNA

We searched the second data set for additional cases of ctDNA, with a distinct genotype appearing because of *de novo* treatment. In patient 10, elevated levels of ctDNA bearing activating and T790M mutations were detected at day 550 (Fig. 2b) after radiation therapy to treat pubic metastasis. This elevation was distinct from other ctDNA elevations that related to ctDNA bearing only the activating mutation, such as the peak upon the initial disease progression (day 325) and the peak that appeared during treatment with gefitinib and pemetrexed (day ~ 690). This peak was likely owing to radiation-induced destruction of cancerous tissue carrying both activating and T790M mutations, and its corresponding ctDNA may be classified as T-type ctDNA.

## Discussion

**Figure 3:**
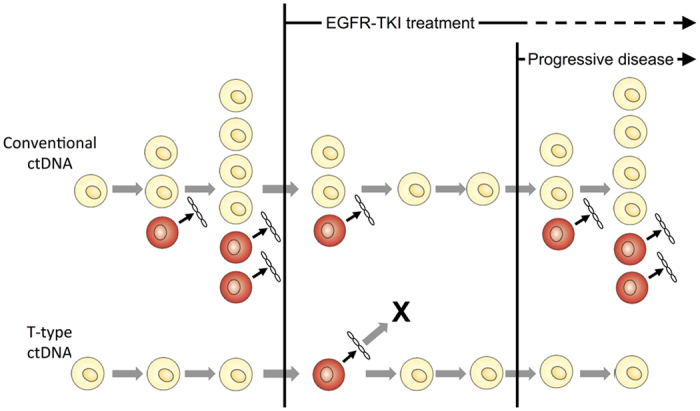
Schematic representation of conventional and T-type ctDNAs in the EGFR-TKI treatment. Yellow cells represent living cancer cells, whereas red cells represent dead cancer cells that release ctDNA. The abundance of conventional ctDNA increase as cancer grows; however, it decreases to undetectable levels upon initiation of the EGFR-TKI treatment, increases again upon disease progression owing to acquiring resistance. In contrast, T-type ctDNA appears upon initiation of EGFR-TKI, then disappears, and does not reappear during the observation period. The absence of reemergence may be due to a complete elimination of this subpopulation by EGFR-TKI treatment, or the subpopulation may persist without producing detectable ctDNA amounts.

The diagnostic limitation of ctDNA analysis is its inability to detect cancer cell populations with few or no dying cells. In contrast, serum biomarkers used for cancer diagnosis are released from living cells. In this study, we found that ctDNA with a genotype different from that of the major cancer cell population appeared upon initiation of EGFR-TKI or radiation therapy, then disappeared and did not appear again during the observation period. This ctDNA, termed T-type ctDNA, originates from cancer cell subpopulations that normally exhibit very low proportions of dying cells; however, they are killed during therapy and release ctDNA. Our observations suggest that *de novo* treatment can reveal the existence of cancer cell subpopulations normally not detectable by ctDNA analysis. In contrast, conventional ctDNA represents cancer cell populations actively proliferating and renewing themselves, while maintaining a certain proportion of dying cells. The dynamics of cancer cell populations undergoing EGFR-TKI treatment are schematically represented in Fig. 3. Although we focused on T-type ctDNA with a genotype that was not identified in biopsy samples, T-type ctDNA with the genotype found in biopsy samples may represent a similar cancer cell subpopulation.

Cancer cell subpopulations detected by T-type ctDNA may be related to cancer dormancy^3^,^7^. For example, recurrence after more than 10 years of latency is not uncommon among breast cancer patients^8^. Dormant cancer cells may be under G0/G1 arrest, therefore, resistant to chemotherapy, because cytotoxic agents only affect proliferating cells. In contrast, inhibition of EGFR disrupts signaling pathways involved in cell survival and can kill dormant cells^9^. The absence of T-type ctDNA upon initiation of chemotherapy might be explained either by cancer dormancy or due to complete elimination of the corresponding cancer cells by the preceding EGFR-TKI treatment. Another possibility is that the assays may have missed the appearance of T-type ctDNA, because T-type ctDNA only appears transiently.

Another explanation could be that cancer subpopulations releasing T-type ctDNA are not dormant; rather, they exhibit infrequent renewal, that is, a low turnover rate of cancer cells. The pubic metastasis described above could contain such subpopulations. It should be noted that the current views on proliferation and survival of cancer cells, including the concept of dormancy, are hypothetical. The existing experimental data are inadequate to link them with the clinical observations. Thus, the aforementioned proposed characteristics of the populations that release T-type ctDNA are speculative; further studies are necessary to identify the biological properties of subpopulations represented by T-type ctDNA.

This study suggests that ctDNA with an activating mutation not identified in biopsy samples is not uncommon. Combining the first and the second data sets, ctDNA with this mutation pattern appeared in 18.3% (11 in 60) of the patients. This figure is considerably higher than the incidence of the exon 19 deletion/L858R double mutations in biopsy samples, which is reported as 2.1–3.6%^10^,^11^,^12^. Experimental artifacts are unlikely, because the false positive rates of the assay system are low, and the probability of segregation of the T-type ctDNA in the limited period is low. One possibility is the presence of intratumor heterogeneity that cannot be detected with the routine *EGFR* mutation test. A previous study on lung adenocarcinomas^13^ indicated that although intratumor heterogeneity was not found with exome sequencing (average depth of 277×), it was often found with extensive deep sequencing (average depth of 863×). This study concluded that a considerable sequencing depth is necessary to detect cancer gene mutations and accurately characterize the intratumor heterogeneity of lung adenocarcinomas.

It is also important to note that analysis of spatial and temporal genetic heterogeneity of *EGFR* mutations in lung cancer tissues is sparse. A case report with data from a long-term observation demonstrated the existence of two activating mutations in the same patient, at different time points^14^. Extensive analysis of primary and metastatic lesions is required to confirm the heterogeneity found in this study. Currently, ctDNA assays are being developed using somatic mutations based on mutation screening of primary tumors. Such assays would neglect important information derived from the genetic heterogeneity of cancer cell subpopulations in a patient.

In the first data set, the detection rate of ctDNA before EGFR-TKI treatment was 66.7% (14 out of 21 patients), which is in consensus with the data established by various studies^6^,^15^,^16^; however, it increased to 85.4% (18 out of 21) when the transient peaks after initiation of treatment were taken into account. Conventional ctDNA decreases upon initiation of new therapies, whereas T-type ctDNA appears after the initiation of the EGFR-TKI treatment. Thus, performing assays before and after the initiation would increase the detection rate. This principle may be applied to the resistant mutation, T790M. Identification of T790M for the application of novel EGFR-TKIs such as AZD9291^17^ and rociletinib^18^ is indispensable; however, there is discordance in T790M detection between biopsies and plasma samples^19^. Performing assays before and after interventions with cytotoxic agents and radiation may improve the detection rates of T790M. Analysis of T-type ctDNA can provide data on tumor heterogeneity^20^. Timing of blood sampling should also be carefully determined, because of the transient presence of T-type ctDNA.

T-type ctDNA highlights the presence of cancer cell populations normally not detected through ctDNA analysis in advanced cancer. However, cancer cell populations releasing T-type ctDNA may commonly be found in early stage cancers. The low detection rate of ctDNA in early stage cancer^21^ may be due to the existence of such populations. Since intervention in normal individuals is not practical, early detection of cancer cells with similar characteristics would be difficult, indicating a biological limitation of ctDNA in early detection.

## Materials and Methods

### Patients

Two previously obtained data sets were used in this study. The first data set had originally been obtained for analysis of ctDNA appearing at early stages of EGFR-TKI treatment (University Hospital Medical Information Network Clinical Trials Registry UMIN000006764)^22^. The patients were prospectively recruited from August 2013 to April 2014 from the Osaka Medical Center for Cancer and Cardiovascular Diseases. Patients were subjected to their first EGFR-TKI monotherapy or their first combinatorial therapy with EGFR-TKI and cytotoxic agents, regardless of any prior therapy. The blood-sampling schedule was as follows: (1) within 1 week before the initiation of therapy, (2) 2 or 3 days after initiation, (3) 8 days after initiation, and (4) 15 days after initiation. A leeway of ±2 days (not counting holidays) was allowed for all samples collected after initiation (2, 3, and 4). CT scans were performed within 28 days before treatment initiation.

The second data set was based on long-term observation of ctDNA; these data were originally obtained for use in a study that examined the correlation between ctDNA and disease progression^4^,^23^. Lung cancer patients with *EGFR*-activating mutations being subjected to EGFR-TKI treatment for the first time, regardless of having received any other treatments, were prospectively recruited (University Hospital Medical Information Network Clinical Trials Registry UMIN000006764) between November 2011 to March 2014 from the Osaka Medical Center for Cancer and Cardiovascular Diseases. Blood sampling was performed before the initiation of EGFR-TKI treatment as well as two and four weeks (14 and 28 days) after initiation. A leeway of ±4 days (not counting holidays) in sampling times was allowed. Further samples were to be collected every two months, until at least the onset of objective disease progression and beyond this point if possible. Data from patients who had been included in the first data set were removed from the second set.

Written informed consent was obtained from all participants. This study was approved by the ethics committee of the Osaka Medical Center for Cancer and Cardiovascular Diseases. The assays were carried out in accordance with the approved guidelines.

### DNA extraction from plasma samples

Plasma was prepared via centrifugation of 4–5 ml of EDTA-treated blood at 800 *g* for 10 min at room temperature. The plasma was transferred to a fresh tube and re-centrifuged at 15,100 *g* for 10 min at room temperature. After centrifugation, the upper plasma was transferred to a fresh tube. Centrifuged plasma samples were frozen at −80 °C until DNA extraction. DNA was extracted from 1.5–2.0 ml of a plasma sample using the QIAamp circulating nucleic acid kit (Qiagen, Hilden, Germany) according to the manufacturer’s instructions.

### Amplicon library construction and deep sequencing

Positions of PCR-target regions and primer sequences were previously described^24^. PCR amplification was conducted in a 50 μl reaction mixture containing plasma DNA obtained from 300 μl of plasma (approximately 10 ng), 20 pmol of each primers and 1 unit of KOD -Plus- DNA polymerase (Toyobo, Osaka, Japan). The cycling profile was as follows: 2 min at 94 °C for initial denaturation, followed by 40 cycles of 15 sec at 94 °C for denaturation, 30 sec at 55 °C for annealing, and 50 sec at 68 °C for extension. The products were purified using the QIAquick 96 PCR Purification Kit (Qiagen) or the MinElute PCR Purification Kit (Qiagen). Subsequently, we mixed equal amounts of the purified PCR products and diluted them to create a template for emulsion PCR. We mixed 12 and 24 types of PCR products for use with Ion 318 semiconductor chips (Thermo Fisher Scientific, Waltham, MA, USA), respectively.

Sequencing template preparation was carried out using an Ion OneTouch Template Kit (Thermo Fisher Scientific) and Ion OneTouch system (Thermo Fisher Scientific) according to manufacturer’s protocol. Prepared templates were sequenced using Personal Genome Machine^25^ (Thermo Fisher Scientific) with the latest version of reagents.

Deep sequencing with molecular barcodes was performed using the non-overlapping integrated read sequencing system^26^.

### Counting variants

Reads in FASTQ files were divided using 5-nt indexes for individual assignment using in-house perl script. Short reads (<70 bases) were discarded. Remaining reads were aligned to target sequences (exon 19, 20 and 21 of EGFR gene) with bwa (version 0.6.2) using the bwasw mode for aligning long reads^27^ and parameter setting “-b5 -q2 -r1 -z10”.

Using samtools (version 0.1.18)^28^, the generated mapping data by bwa (SAM files) were converted to BAM files and processed to obtain the per base coverage (pileup files). Subsequently, we summarized the base counts for each target base position (e.g., EGFR codons 790 and 858) using an in-house-devised perl script. Frequencies of variants/errors (substitutions) were calculated by dividing base counts of substitutions by all base counts on each position. Because of a high error rate for insertions/deletions, detection of several base-pair deletions in exon 19 was difficult. Instead, we aligned reads to ten template sequences corresponding to major deletion types using bwa. The nucleotide positions for these deletions in the human genome (GRCh37/hg19) are 55242465-55242479, 55242466-55242480, 55242468-55242482, 55242469-55242486, 55242469-55242478, 55242469-55242481, 55242470-55242487, 55242467-55242485, 55242467-55242481, 55242467-55242475 in chromosome 7. The frequencies of the deletion mutations were estimated by dividing the number of reads aligned to deletion-type sequences by the number of all reads aligned to exon 19 sequences with or without a deletion. Because incomplete matches with deletion-type template sequences were observed due to the diversity of deletions^29^, we employed the deletion type with the maximum number of aligned reads for the estimation of mutation frequency.

### Output of the assay system

A diagnostic score, termed the plasma mutation (PM) score, was defined as the number of reads with deletions (exon 19 deletions) or substitutions (exon 20: T790M; exon 21: L858R and L861Q) per 100,000 reads. We deduced parameters corresponding to the limit of detection (LOD) and the limit of quantification (LOQ)^24^ and used them to define a threshold for mutation detection. The LOD is defined as the lowest concentration or amount of an analyte that can be reliably identified as qualitatively present in a sample. The LOQ is defined as the lowest concentration or amount of an analyte that can be reproducibly quantified in a sample. In accordance with a previous study^6^, the threshold for ctDNA with the activating mutation found in biopsy samples was set as LOQ for exon 19 deletion and LOD for L858R. For the ctDNA bearing the activating mutation not identified in biopsy samples and T790M, we chose a conservative approach and set the threshold of detection as LOQ (PM score = 300). The multi-institute study^6^ conducted parallel to the current one deduced that the possibility of false positives was negligible under this setting, as the estimated false-positive rates for exon 19 deletion, L858R, and T790M, were 2%, 0%, and 1%, respectively.

## Additional Information

**How to cite this article**: Kato, K. *et al*. Transient appearance of circulating tumor DNA associated with *de novo* treatment. *Sci. Rep.* **6**, 38639; doi: 10.1038/srep38639 (2016).

**Publisher's note:** Springer Nature remains neutral with regard to jurisdictional claims in published maps and institutional affiliations.

## Supplementary Material

Supplementary Information

## Figures and Tables

**Table 1:** ctDNA with an activating mutation that was not present in biopsy samples.

Patient ID	Age	Sex	Stage	EGFR-TKI	Initial response	Mutation in biopsy samples	Mutation of T-type ctDNA	Time point of T-type ctDNA*	Time point of objective disease progression*	Time point of last sample*	Therapies after disease progression
E5	65	F	IV	Gefitinib	PR	Exon 19 deletion	L858R	2	—	19	NA
E6	67	M	IV	Gefitinib	PR	L858R	Exon 19 deletion	12 19	—	131	NA
E7	76	F	IV	Gefitinib	SD	L858R	Exon 19 deletion	6	—	222	NA
E8	68	F	IIIB	Erlotinib	SD	Exon 19 deletion	L858R	7	—	70	NA
1	77	F	IIIA	Erlotinib	PR	L858R	Exon 19 deletion	28	659	1115	Erlotinib (continuation)
2	39	F	IV	Gefitinib	PR	Exon 19 deletion	L858R	14	372	436	Erlotinib
3	80	M	IV	Gefitinib	PR	L858R	Exon 19 deletion	347	296	440	CBDCA
5	69	F	IV	Gefitinib	PR	L858R	Exon 19 deletion	14	383	475	CDDP+PEM
13	66	F	IIIA	Gefitinib	NE	L858R	Exon 19 deletion	28	217	283	Erlotinib
15	56	F	IIIB	Gefitinib	PR	L861Q	Exon 19 deletion	34	335	761	CDDP+PEM
K192	73	F	IV	Gefitinib	PR	L858R	Exon 19 deletion	30	—	747	NA

E5 - E8, the first data set; 1 - K192, the second data set; ^*^, days after EGFR-TKI initiation. Patient IDs of the second data set are those used in a previous publication^23^ except K192. Abbreviations are as follows: PR, partial response; SD, stable disease; NE not evaluable; NA, not applied; CBDCA, carboplatin; CDDP, cisplatin; PEM, pemetrexed.
