# Supplementary material for: Transient appearance of circulating tumor DNA associated with de novo treatment
Source: Sci Rep. 2016 Dec 9;6:38639. doi: 10.1038/srep38639 (PMC5146655; doi:10.1038/srep38639)
Supplement: Supplementary Information [file srep38639-s1.pdf]

Supplementary Information

Transient appearance of circulating tumor DNA associated with *de novo* treatment

Kikuya Kato[1], Junji Uchida[2], Yoji Kukita[1], Toru Kumagai[2], Kazumi Nishino[2], Takako Inoue[2], Madoka Kimura[2], and Fumio Imamura[2]

[1]Department of Molecular and Medical Genetics, Research Institute, Osaka Medical Center for Cancer and Cardiovascular Diseases, Osaka, Japan

[2]Department of Thoracic Oncology, Osaka Medical Center for Cancer and Cardiovascular Diseases, Osaka, Japan

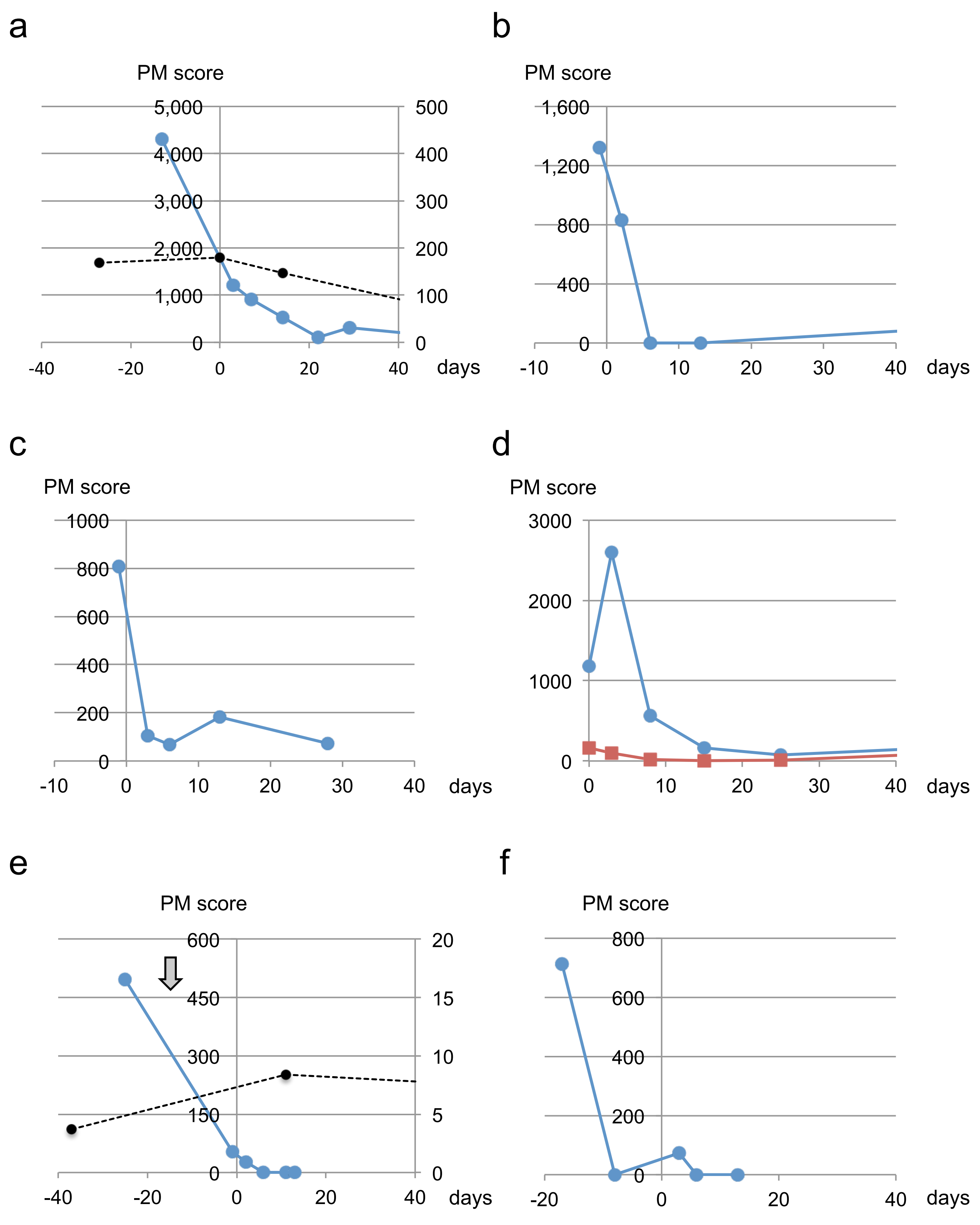
a
PM score
5,000
4,000
3,000
2,000
1,000
0
500
400
300
200
100
0
-40
-20
0
20
40
days
b
PM score
1,600
1,200
800
400
0
-10
0
10
20
30
40
days
c
PM score
1000
800
600
400
200
0
-10
0
10
20
30
40
days
d
PM score
3000
2000
1000
0
0
10
20
30
40
days
e
PM score
600
450
300
150
0
20
15
10
5
0
-40
-20
0
20
40
days
f
PM score
800
600
400
200
0
-20
0
20
40
days

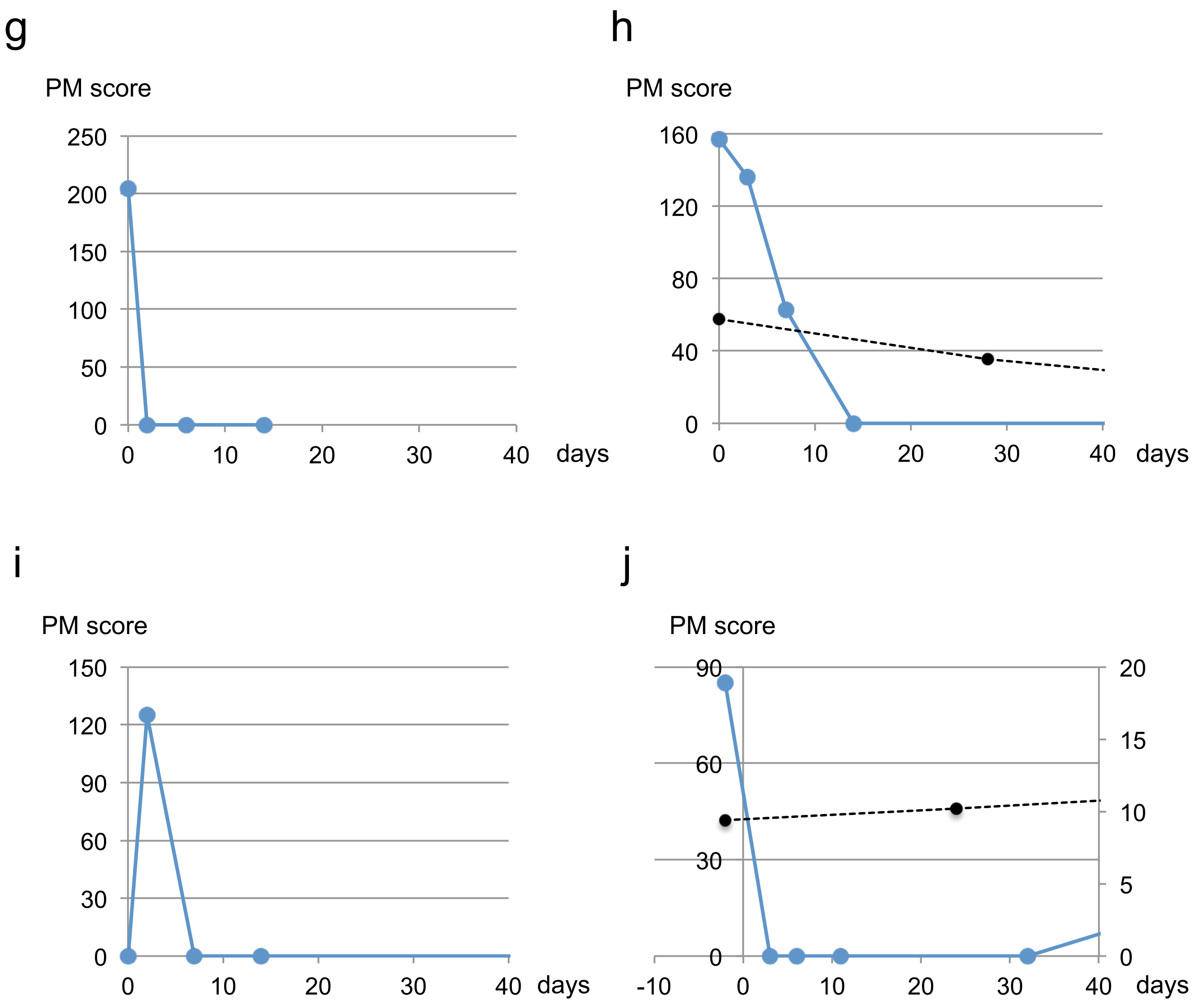

Supplementary Figure S1. ctDNA dynamics in the early period of the EGFR-TKI treatment. These patients are not represented in Figure 1. a. Patient E9; b. Patient E10; c. Patient E11; d. Patient E12; e. Patient E13; f. Patient E14; g. Patient E15; h. Patient E16; i. Patient E17; j. Patient E18. The details of the panels are the same as those in Figures 1 and 2.

Supplementary Table S1. Clinical information of patients included in the first data set.

Patient_ID	Age	Sex	Stage	EGFR-TKI	Initial response	Activating mutation	Therapies before EGFR-TKI
E1	81	F	IV	Erlotinib	PR	L858R	Radiation
E2	54	F	IV	Gefitinib	PR	L858R	None
E3	69	M	IV	Gefitinib	PR	Exon 19 deletion	Radiation (bone), CBDCA+PTX +AMG706
E4	56	F	IIA	Gefitinib	NE	Exon 19 deletion	Curative surgery, CDDP+VNR
E5	65	F	IV	Gefitinib	PR	Exon 19 deletion	Radiation (brain)
E6	67	M	IV	Gefitinib	PR	L858R	None
E7	76	F	IV	Gefitinib	SD	L858R	None
E8	68	F	IIIB	Erlotinib	SD	Exon 19 deletion	CBDCA+PTX, Radiation
E9	86	F	IV	Gefitinib	PR	Exon 19 deletion	None
E10	68	F	IV	Gefitinib	PR	Exon 19 deletion	None
E11	62	F	IV	Erlotinib	PR	L858R	None
E12	75	F	IIIA	Erlotinib	PR	L858R	Radiation (brain)
E13	66	M	IV	Erlotinib	NE	Exon 19 deletion	Radiation (bone)
E14	65	F	IV	Erlotinib->gefitinib	PR	L858R	None
E15	77	F	IB	Gefitinib	NE	L858R	Surgery
E16	58	F	IV	Erlotinib	NE	L858R	Radiation (bone)
E17	74	F	IIIA	Gefitinib	PR	L858R	CDDP+VNR, Rdiation
E18	79	F	IV	Gefitinib	SD	L858R	none
E19	77	F	IV	Gefitinib	PR	L858R	Surgery, Radiation
E20	65	M	IV	Gefitinib	SD	L858R	Surgery, Radiation
E21	65	M	IV	Erlotinib->gefitinib	PR	Exon 19 deletion	Radiation

Paitents E19-21 did not have any ctDNA-positive samples.
Abbreviations are as follows: PTX, paclitaxel; VNR, vinorelbine.
